# Comprehensive Analysis of Interactions between Human Serum Albumin and Human Cystatin C – Two Proteins Present in Body Fluids

**DOI:** 10.1021/acsomega.5c04317

**Published:** 2025-08-07

**Authors:** Adriana Żyła, Igor Zhukov, Michał Taube, Daria Wojciechowska, Joanna Wolak, Przemyslaw Jurczak, Paulina Czaplewska, Aneta Szymańska, Anne Martel, Maciej Kozak

**Affiliations:** † Department of Biomedical Physics, Institute of Physics, Faculty of Physics and Astronomy, 49562 Adam Mickiewicz University, Poznań 61-614, Poland; ‡ 90863 Institute of Biochemistry and Biophysics of the Polish Academy of Sciences, Warsaw 02-106, Poland; § Department of Biomedical Chemistry, Faculty of Chemistry, University of Gdańsk, Gdańsk 80-308, Poland; ∥ Specialist Laboratories, 540749 Intercollegiate Faculty of Biotechnology of University of Gdańsk and Medical University of Gdańsk, Gdańsk 80-307, Poland; ⊥ Large Scale Structures, ILL Neutrons for Society, 56053 Institute Laue-Langevin, Grenoble 38042, France; # SOLARIS National Synchrotron Radiation Centre, Jagiellonian University, Kraków 31-007, Poland

## Abstract

Human cystatin C (HCC) and human serum albumin (HSA) are proteins that coexist in body fluids. The main physiological role of HCC is the inhibition of cysteine proteases, while HSA is a universal transport protein carrying numerous ligands. Both proteins are also associated with amyloidogenesis at various levels, so understanding their potential interactions is an important aspect of the research. To characterize these interactions, we used several combined complementary techniques: microscale thermophoresis (MST), isothermal titration calorimetry (ITC), high-resolution mass spectrometry (HR-MS), and nuclear magnetic resonance spectroscopy (NMR). The *K*_D_ value obtained from the MST method was 1.3 μM, while the dissociation constant determined by ITC was 1.15 μM. Mass spectrometry data confirmed formation of the HSA–HCC complex with 1:1 stoichiometry. The most pronounced changes were observed in the NMR spectrum for the prolonged dynamic processes, characterized by increased spectral densities for several HCC residues.

## Graphical abstract

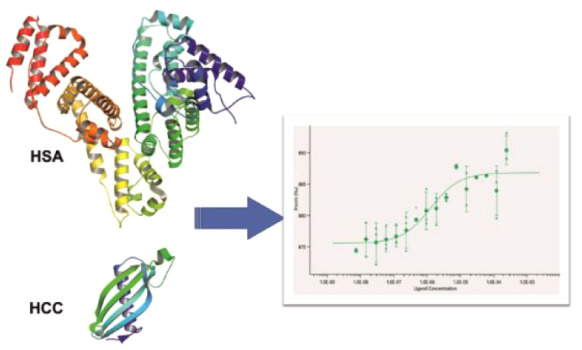

## Introduction

Recent progress in experimental structural biology (protein crystallography,^1,2^ cryo-electron microscopy,^3^ and nuclear magnetic resonance spectroscopy^4^) complemented by significant progress in artificial intelligence-driven methods for predicting and simulating protein structures, has opened up enormous opportunities for structural biology. By leveraging existing structural models and the use of methods based on artificial intelligence,^5^ it is possible to obtain structural models of an extensive array of new proteins based solely on their amino acid sequences (by using AlphaFold2 ^7^), while AlphaFold3 ^6^ brings us closer to protein–protein interaction prediction. However, despite such substantial progress in the field of structural biology, particularly in structure prediction, the accurate prediction of macromolecular complex formation and the mapping of intermolecular interactions remain challenging. Therefore, the experimental characterization of the physiologically relevant patterns of protein–protein interactions within the human body is essential for a comprehensive understanding of various biological processes, including those occurring in body fluids.

Among various body fluids, blood plasma (serum) and cerebrospinal fluid (CSF) together constitute approximately one-fourth of the extracellular fluid volume in the human body. Therefore, the analysis of their composition, including monitoring fluctuations in the concentration of their components and intermolecular interactions, in particular with regard to biomarkers, is extremely valuable from the point of view of medical diagnostics^8^ or drug pharmacokinetics.^9^

Blood plasma is the main fraction of blood, representing approximately 55% of its volume and containing electrolytes, proteins, and nutrients. Approximately 7% of the blood plasma volume is composed of proteins, with human serum albumin (HSA) being the most abundant. Cerebrospinal fluid, which is responsible for protecting the central nervous system, is, in fact, a filtrate of blood plasma; thus, its composition is similar, although some differences have been observed.^10^ Specifically, CSF contains up to 0.6% proteins,^11^ with albumin also identified as one of the main components.^10^

Human serum albumin (HSA) has the ability to bind numerous ligands, which makes it a storage and transporter molecule for numerous endogenous and exogenous substances - from metal ions^12^ and small molecules^13^ to peptides.^14^ Structurally, HSA resembles a heart and consists of three main domains (I–III), which can be further divided into two parts (A and B), forming a total of six functional domains, each with different binding capabilities toward various ligands (for review, see the report by Mishra and Heath^15^). The structure of human serum albumin,^16^ obtained by our group using cryo-EM microscopy, is presented in Figure 1.

**Figure 1:**
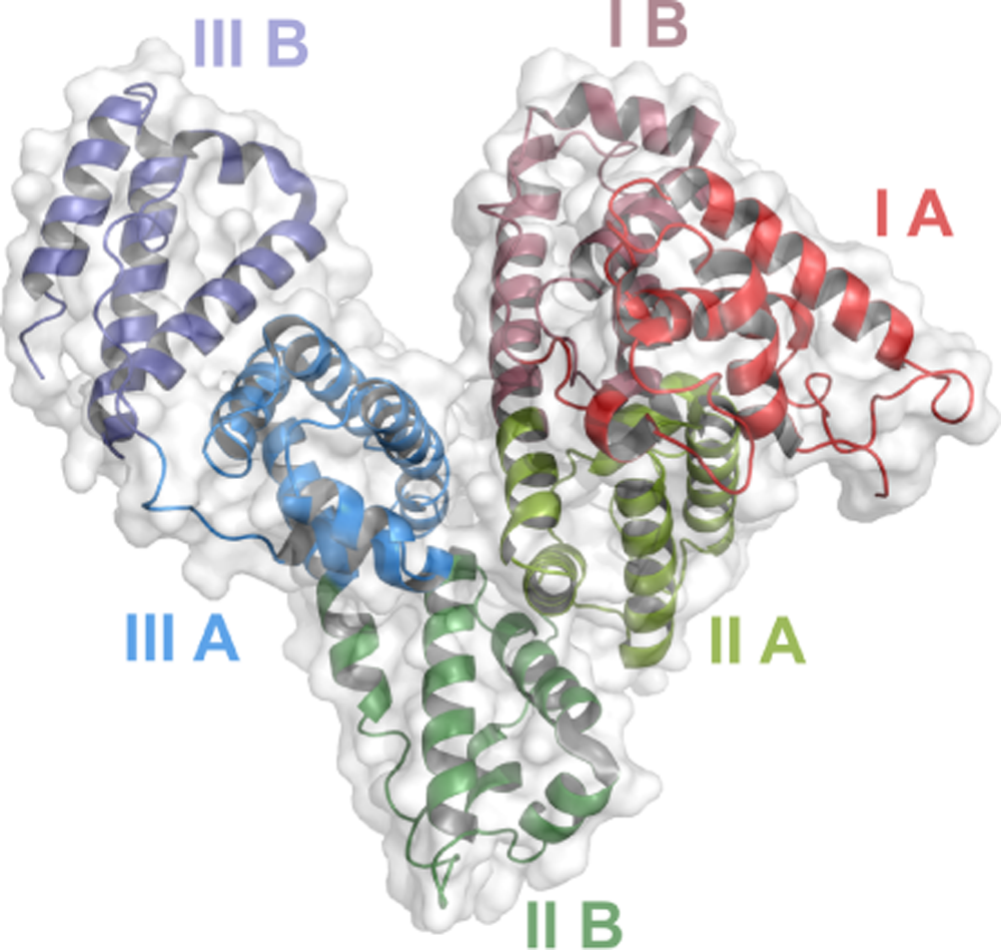
Human serum albumin. The structure of the fatty acid-free form obtained by cryo-electron microscopy at a resolution of 0.38 nm (PDB code: 8Q3F^16^). The figure was colored according to the domain structure indicated in the text. The image was generated by using PyMol (www.pymol.org).

Recent studies have shown that human serum albumin not only serves as a transporter for ions and small molecules but also forms complexes with various proteins. The set of proteins interacting with HSA is referred to as the albuminome.^17^ Most of the interactions between HSA and other proteins remain functionally uncharacterized. Two notable examples are the interaction of albumin with cell surface receptors and the amyloid beta (Aβ) peptide. Several cell surface receptors bind albumin and facilitate its endocytosis for turnover and ligand uptake.^18^ Moreover, it has been shown that HSA can bind Aβ peptides and regulate Aβ fibril formation in vitro.^19^ Considering the overall protein concentration in the plasma, there is a chance that low-affinity and low-abundance proteins can form complexes with HSA because their effective concentration is enhanced due to the exclusion volume effect.^20^

In addition to HSA, human cystatin C (HCC) is another protein that is present in both plasma and cerebrospinal fluid and is the tenth most abundant protein in urine. HCC is a small globular protein (13.3 kDa) that functions in the body as an inhibitor of cysteine proteases from the papain and legumain families.^21^ The efforts to solve the structure of this protein led to one of the first observations of the domain-swapping phenomenon accompanying the formation of dimers of native HCC.^22–24^ The structure of the monomeric form of HCC, depicted in Figure 2, was resolved only for its point mutants^21,22^ that stabilize the monomeric form. The pathogenic point mutation in the HCC sequence (L68Q) is associated with the development of hereditary cystatin C amyloid angiopathy (HCCAA), a serious disease that results in the death of patients aged 15 to 45. This mutation greatly accelerates fibril formation and their accumulation in the walls of arteries of the central nervous system, leading to numerous cerebral hemorrhages.^25^ However, studies performed on a mouse model show that cystatin C binds amyloid-beta and inhibits fibril formation.^26^

**Figure 2:**
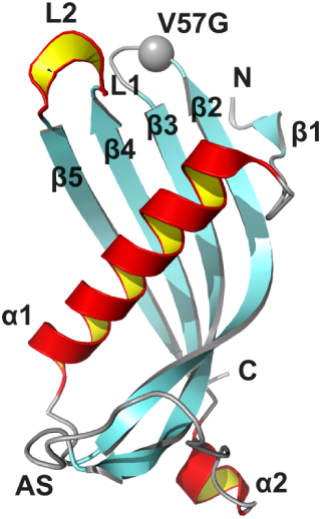
Human cystatin C: structure of the monomeric form of HCC with the point mutation V57G (indicated as a gray sphere) (PDB code: 6ROA^27^). The figure was colored according to secondary structure elements (red – α-helices, cyan – β-sheets, gray – loops). N and C indicate the protein termini, and AS is a fragment called “appending structure”, unrelated structurally to the core of the fold. The image was generated using PyMol (www.pymol.org).

The structure of the monomeric form of HCC is characterized by the presence of two α-helices, one short and one elongated, lying upon the five-stranded antiparallel β-sheet. The structure is stabilized by the two disulfide bridges, with the loops between β-strands playing a crucial role in interactions with proteases. Human cystatin C is commonly used as a biomarker for detection of kidney dysfunction,^28,29^ in particular the so-called shrunken pore syndrome.^30^ Furthermore, there are reports indicating the potential use of HCC in conjunction with HSA as biomarkers to differentiate diseases in patients with primary chronic glomerulonephritis (PCG) and hypertension-induced renal injury (HRI).^31^ It was also reported that in the pathological state of kidney disease, which is also connected with the occurrence of the dimeric form of HCC,^32^ the homeostasis of body albumins is disrupted as well.^33^ Both proteins are often found in the same body fluids but in significantly different concentrations. It is therefore possible that they can form weak complexes, but their stoichiometries and structures remain unknown. Taking into account that both proteins are implicated in amyloidogenesis to varying extents, the investigation of their potential interactions holds very high scientific potential. To elucidate these interactions, a multifaceted approach utilizing complementary techniques was employed, including microscale thermophoresis (MST), isothermal calorimetry (ITC), high-resolution mass spectrometry (HR MS), and nuclear magnetic resonance spectroscopy (NMR).

## Results

Human serum albumin (HSA) and human cystatin C (HCC) coexist in the same body fluids, such as plasma and CSF. Considering an analogy to amyloid beta (Aβ), which, similarly to HCC, is known to self-associate into oligomers and fibrils, and whose processes can be modulated by interaction with HSA, we sought to investigate the potential interactions between HSA and HCC. In the initial phase of our study, we aimed to confirm the existence of interactions between these two proteins. Subsequently, we intended to characterize the nature of this interaction at the molecular level.

### Determination of Interactions between HSA and HCC by Affinity Chromatography

To investigate the interaction between HSA and HCC, we employed affinity chromatography. Albumin was deposited on a solid support, and HCC served as a ligand. Subsequent analysis of the elution fraction (see Materials and Methods) conducted using mass spectrometry (MALDI), indicated the presence of HCC, while the fractions from the control column, as well as the last milliliter of retentate from the column wash, exhibited no detectable levels of HCC. Figure 3 shows the MALDI spectrum for the elution fraction, where the signal at 13341.8584 *m*/*z* corresponds to the HCC [M + H^+^]^+^ ion, and the signal at 6672.2412 *m*/*z* is the doubly charged ([M+2H^+^]^2+^) HCC signal. These results strongly support the conclusion that HCC and HSA can interact and form complexes, justifying further research.

**Figure 3:**
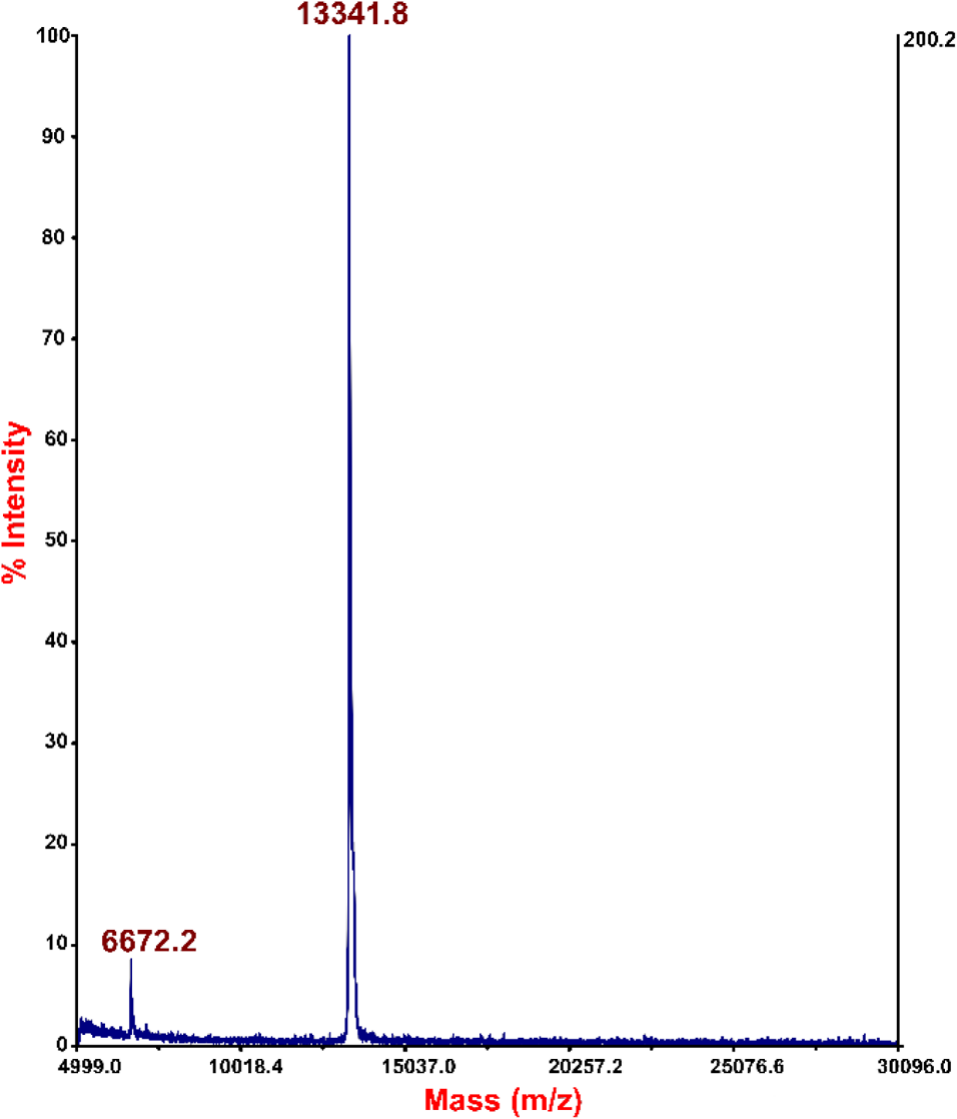
Mass spectrum (MALDI-TOF/MS) recorded for HCC elution from the bed (HSA-bound CNBr-activated Sepharose).

### Analysis of Direct Interactions between HSA and HCC Using Microscale Thermophoresis and Isothermal Titration Calorimetry

The interactions between HSA and HCC were further assessed independently employing the MST (microscale thermophoresis) and ITC (isothermal titration calorimetry) methods. The obtained results are presented in Figure 4A,B, respectively. The dissociation constant, *K*_D_, obtained from the MST technique was 1.3 μM, while the ITC yielded a *K*_D_ value of 1.15 ± 0.01 μM. The results from both methods suggest the formation of an HCC–HSA complex; however, the stability of this complex appears to be relatively low. Further analysis of the ITC data indicated that the interaction between HSA and HCC is primarily driven by the increase of entropy upon binding (the *T*Δ*S* component of Gibbs free energy was −9.99 kcal/mol, and enthalpy was 1.88 kcal/mol). Additionally, the binding stoichiometry, calculated using a single set of identical site binding model, was found to be 0.642. This value is lower than expected for the 1:1 complex. This discrepancy may be attributed to the low signal-to-noise ratio in the measurement.

**Figure 4:**
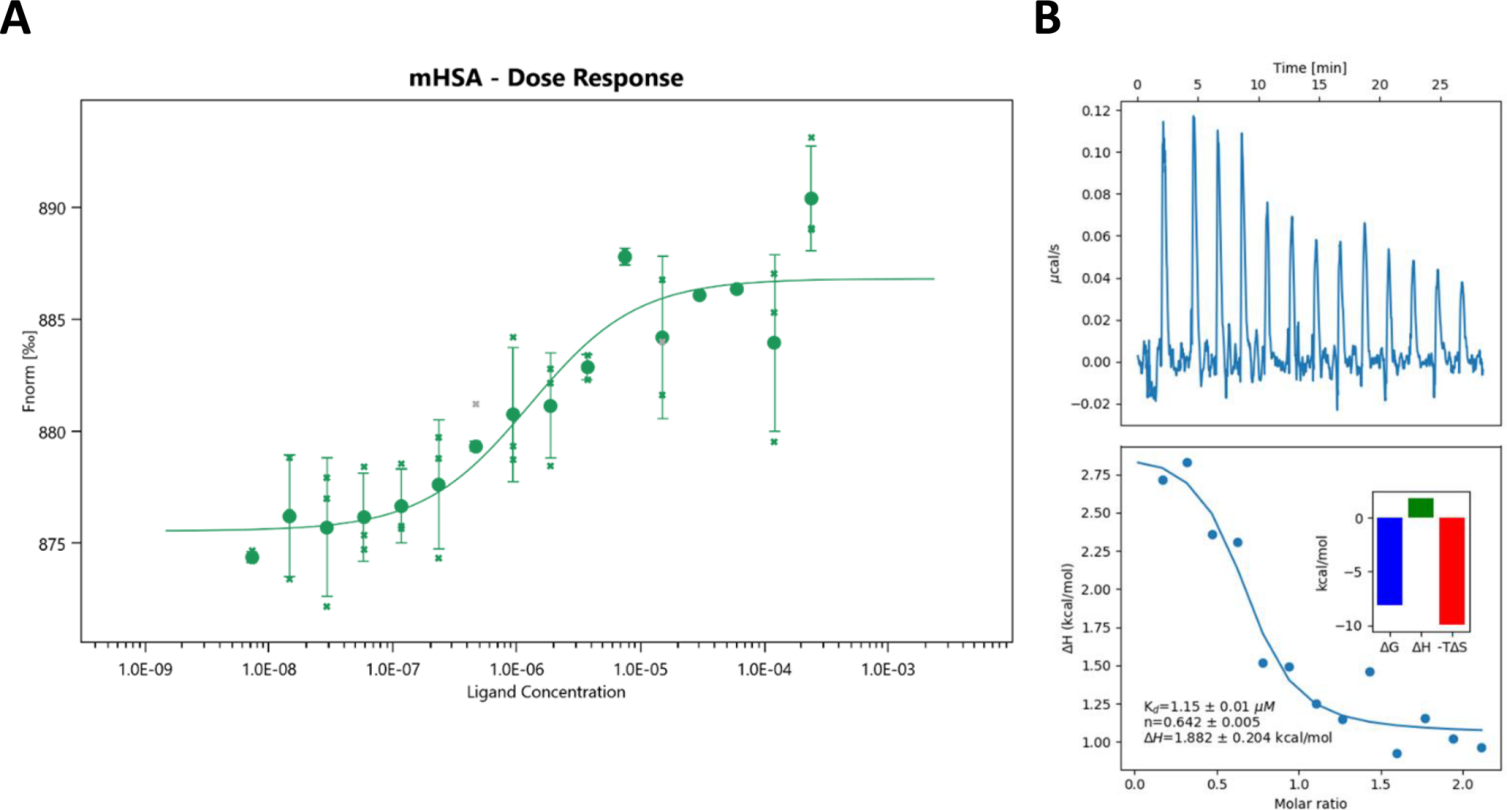
Analysis of purified monomeric HSA (target) and HCC (ligand) interactions by (A) microscale thermophoresis (number of repetitions = 3, error bars - standard deviation) and (B) isothermal microcalorimetry (the ITC curve - top panel; the estimation of binding parameters - bottom panel).

### Characterization of Interaction of HCC with HSA by Mass Spectrometry

In light of the nonstoichiometric binding indicated by the ITC experiment, the next stage of the investigation was to determine the binding ratio in the HSA–HCC complex. The interactions between both proteins were checked in 50 mM ammonium formate at pH 9.0 using native mass spectrometry. HCC and HSA were mixed in a 1:1 molar ratio. The analysis was performed by using mass spectrometry with electrospray ionization in direct injection mode. Subsequent mass reconstruction, conducted based on the obtained spectra, revealed the presence of signals from both ligands as well as the complex (Figure 5). The reconstructed mass of 80 058.4 Da indicates the presence of the complex formed by one molecule of HCC and one molecule of HSA.

**Figure 5:**
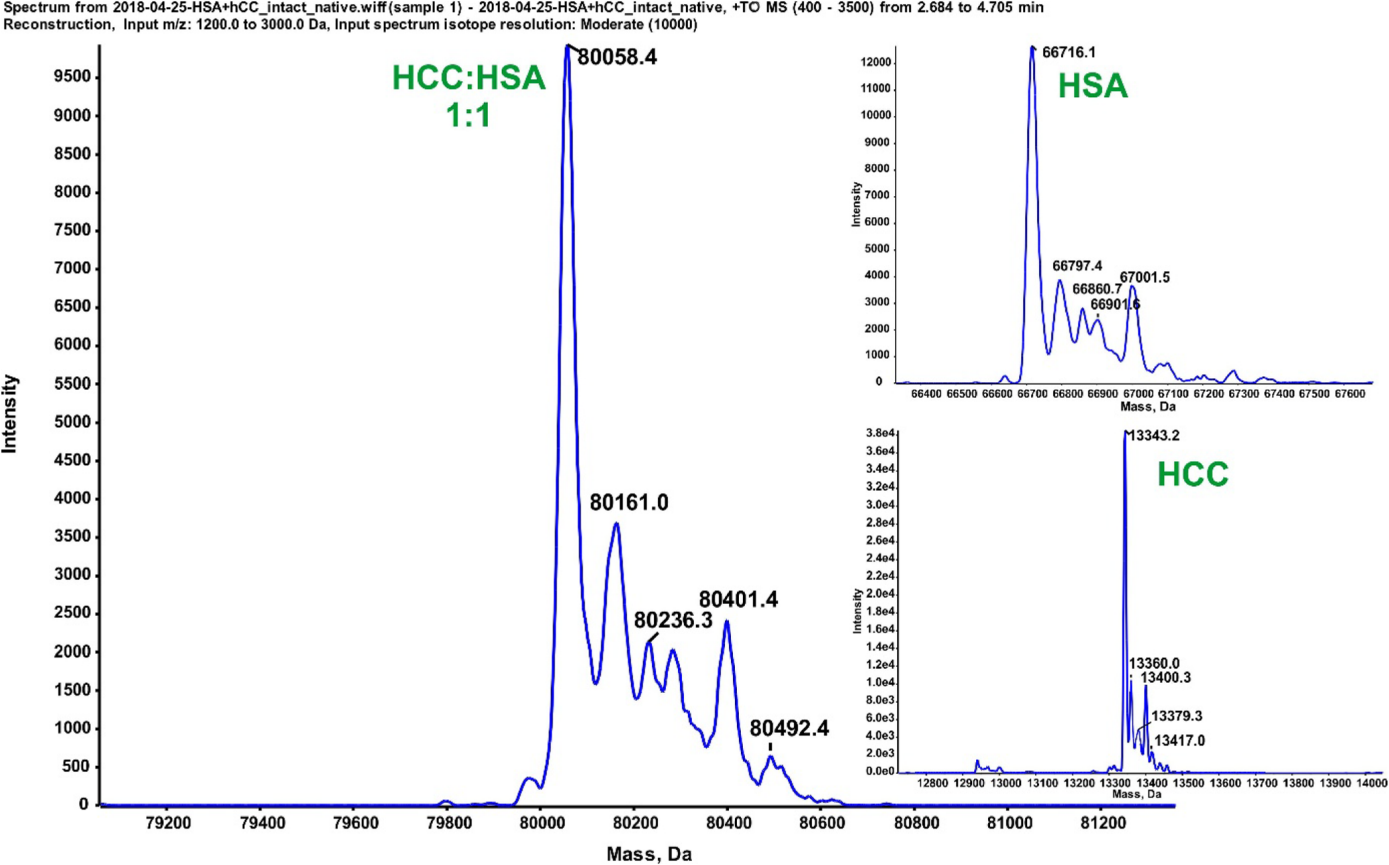
Mass reconstruction of the complexes obtained from the intact mass spectrum of the HSA–HCC mixture in solution. The reference spectra of HSA and HCC are presented in insets.

### NMR Studies of HSA and HCC Interactions

The V57G mutant of HCC is characterized by high conformationally stability and a negligible propensity toward dimerization^22,34^ and formation of higher-mass oligomers,^27^ thus enabling, in contrast to the wild-type molecule, prolonged experiments under NMR technique conditions. For the present study, the V57G HCC was prepared both in the absence and the presence of HSA under exactly the same conditions, thereby allowing for a side-by-side comparison of two sets of experimental data. The collected 2D ^1^H–^15^N HSQC spectra of the uniformly ^15^N-labeled V57G HCC demonstrated no visible shifts in the position of the signals originating from various amide groups of HCC (Figure 6), indicating that the 3D structure of the V57G HCC mutant does not change upon the addition of equimolar amounts of HSA.

**Figure 6:**
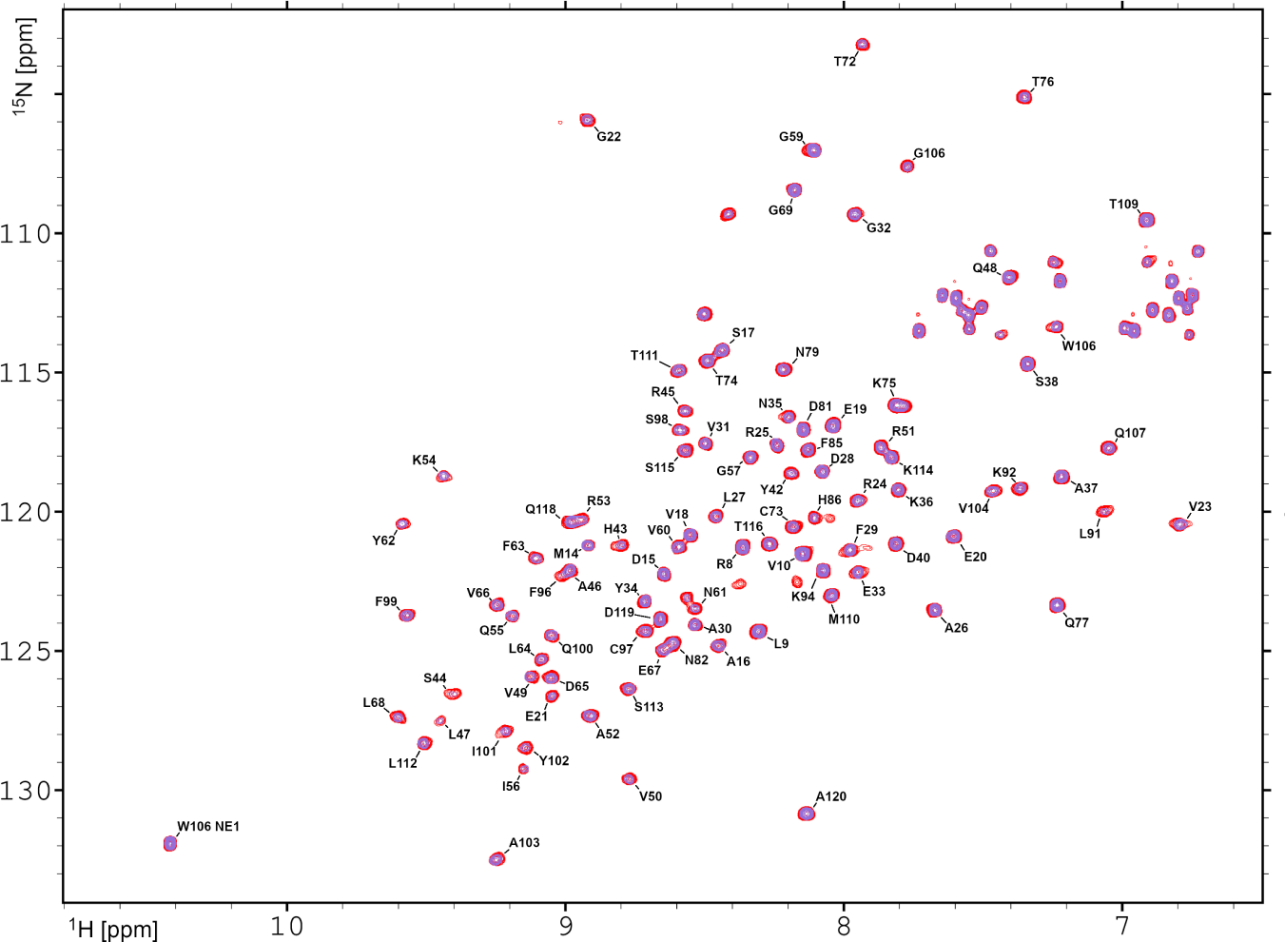
Overlay of the ^1^H–^15^N HSQC spectra collected at 293 K for the uniformly ^15^N-labeled V57G HCC without (red) and with (violet) HSA addition. The sequence-specific assignments of the backbone ^1^H and ^15^N backbone resonances are presented as a one-letter code and sequence number.

To explore the molecular dynamic processes in the HCC backbone, the ^15^N relaxation data – *R*_1_ and *R*_2_ relaxation rates together with ^1^H–^15^N NOE – were acquired for the 81 amide groups out of the 111 expected. The cross-peaks not included in the analysis comprised the disordered, N-terminal HCC fragment consisting of 13 residues (^1^SSPGKPPRLVGGP^13^) and several residues located in the AS (“appending structure”) region exposed to the solvent.^27^ The relaxation experiments performed for V57G HCC provided *R*_1_ and *R*_2_ relaxation rates of 1.081 ± 0.113 (s^–1^) and 15.138 ± 2.641 (s^–1^), respectively (Figure 7). In the presence of HSA in the solution, the relaxation rates were measured as *R*_1_ = 1.025 ± 0.118 (s^–1^) and *R*_2_ = 16.587 ± 2.892 (s^–1^). Comparing the obtained results, we conclude that addition of HSA to HCC in solution leads to an increase in the *R*_2_ relaxation rates, which are usually associated with the dynamic processes occurring at lower frequencies. Small changes in the backbone dynamic processes are further evidenced by the dependence of the local correlation time (τ_C_) calculated for ^15^N in a sequence-specific manner on the *R*_2_/*R*_1_ ratio (Figure 7).^35^ Detailed examination of the calculated τ_C_ revealed that the most pronounced effect of the HSA was observed in the Asp81–Lys92 segment of the V57G HCC mutant.

**Figure 7:**
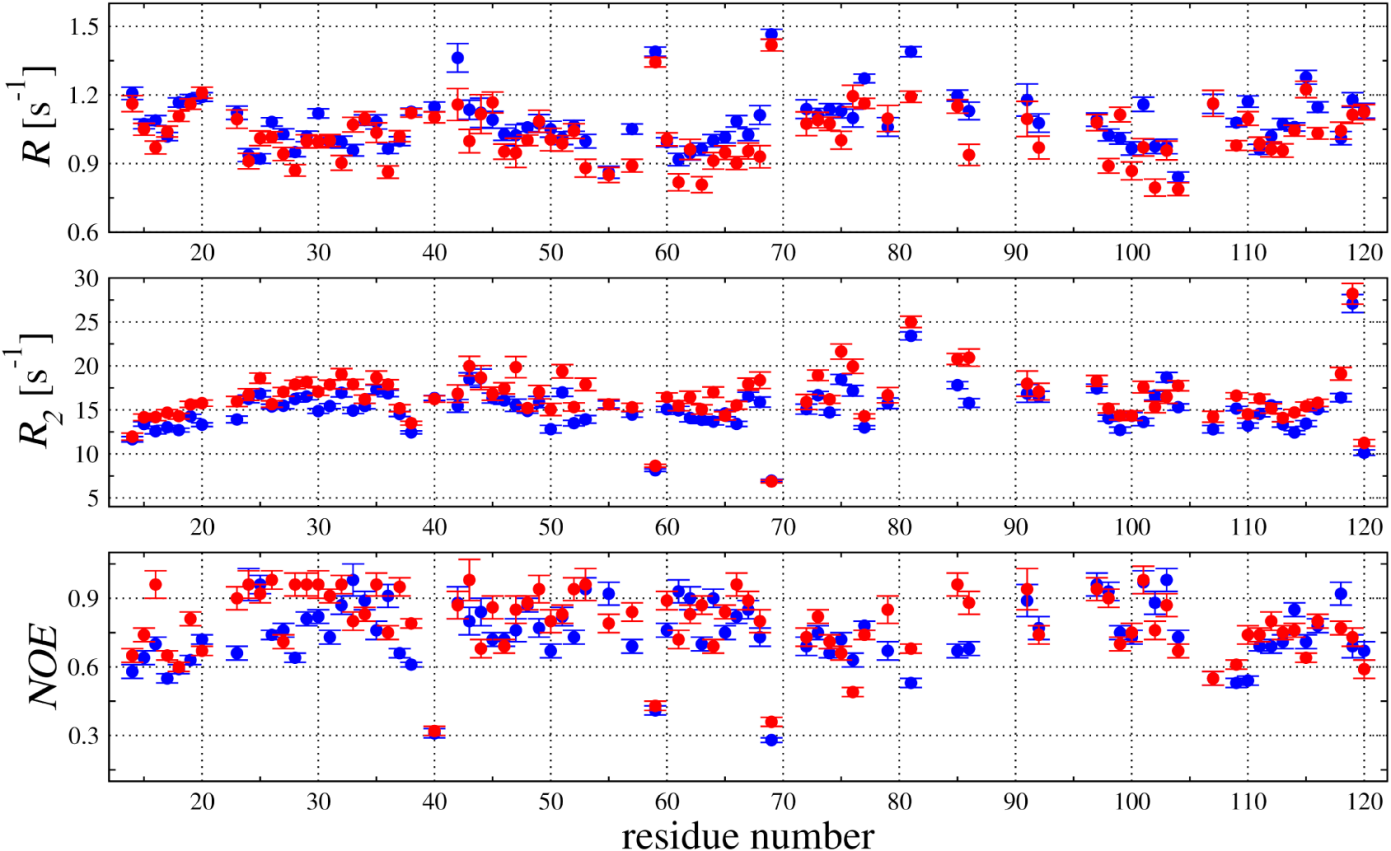
^15^N relaxation parameters (*R*_1_, *R*_2_, and ^1^H–^15^N NOE) acquired at 18.8 T using an Agilent DDR2 800 NMR spectrometer at 298 K. The relaxation parameters obtained in the absence and in the presence of HSA in solution are presented as blue and red, respectively.

The overall correlation time (τ_R_) was calculated on the basis of the *R*_2_/*R*_1_ ratio for the residues in the α-helix and β-sheet, demonstrating that ^1^H–^15^N NOE values higher than 0.75 were 7.45 ± 0.11 (ns) and 8.79 ± 0.03 (ns) for the V57G HCC in the absence and presence of HSA in solution, respectively. The relatively small changes in τ_R_ after the addition of HSA to the solution suggest weak protein interactions. The amount of V57G HCC that interacted with HSA could be estimated on the basis of the following assumption:

τ_R_^–1^ = *p* (τ_R_^f^)^−1^ + (1 – *p*) (τ_R_^b^)^−1^ where τ_R_^f^ is the correlation time of V57G HCC, τ_R_^b^ is the correlation time of V57G HCC bound to HSA, and *p* is a fraction of V57G HCC in solution. Considering the previously defined overall correlation time of HSA as 41 ns,^36^ we estimated the amount of V57G HCC that interacted with HSA as 4% of protein in solution.

Further analysis of the dynamic processes from ^15^N relaxation data was performed by using the SDM approach. The calculated values of the spectral density functions at 0, ω_N_, and 0.87ω_H_ frequencies demonstrated minimal changes between the V57G HCC states with and without HSA in solution (Figure 8).

**Figure 8:**
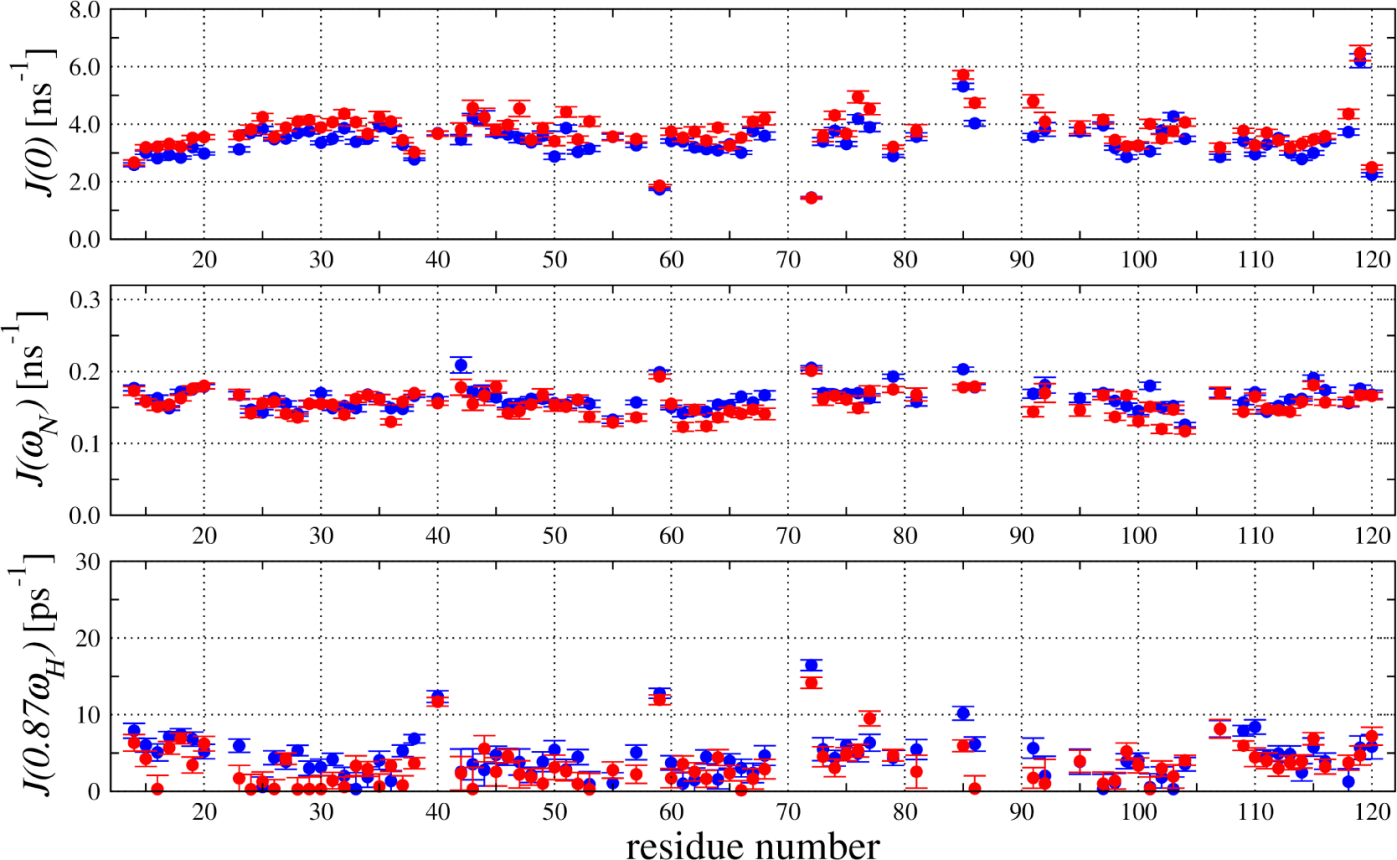
^15^N relaxation data obtained at an 18.8 T magnetic field according to the spectral density mapping (SDM) approach. Spectral densities at *J*(0), *J*(ω_N_), and *J*(0.87ω_H_) evaluated for V57G HCC in the absence and in the presence of HSA are shown as blue and red, respectively.

The most pronounced changes were observed for the prolonged dynamic processes characterized by increased spectral densities for several residues with an *J*(0) dependence. A direct comparison of *J*(0) in the absence and presence of HSA in solution revealed that the majority of the ^15^N nuclei in the amide groups demonstrated the enhancement in the intensity of the molecular dynamic processes at the low-frequency time frame under saturation with HSA (Figure 9A). At the same time, two residues from the L3 loop – Tyr102 and Ala103 – exhibited higher mobility upon interaction with HSA (Figure 9B). Interestingly, Trp106 was in the same β-turn as the residues Tyr102 and Ala103, which suggested an increase in the mobility of the residues in L3 β-turn under interaction with HSA.

**Figure 9:**
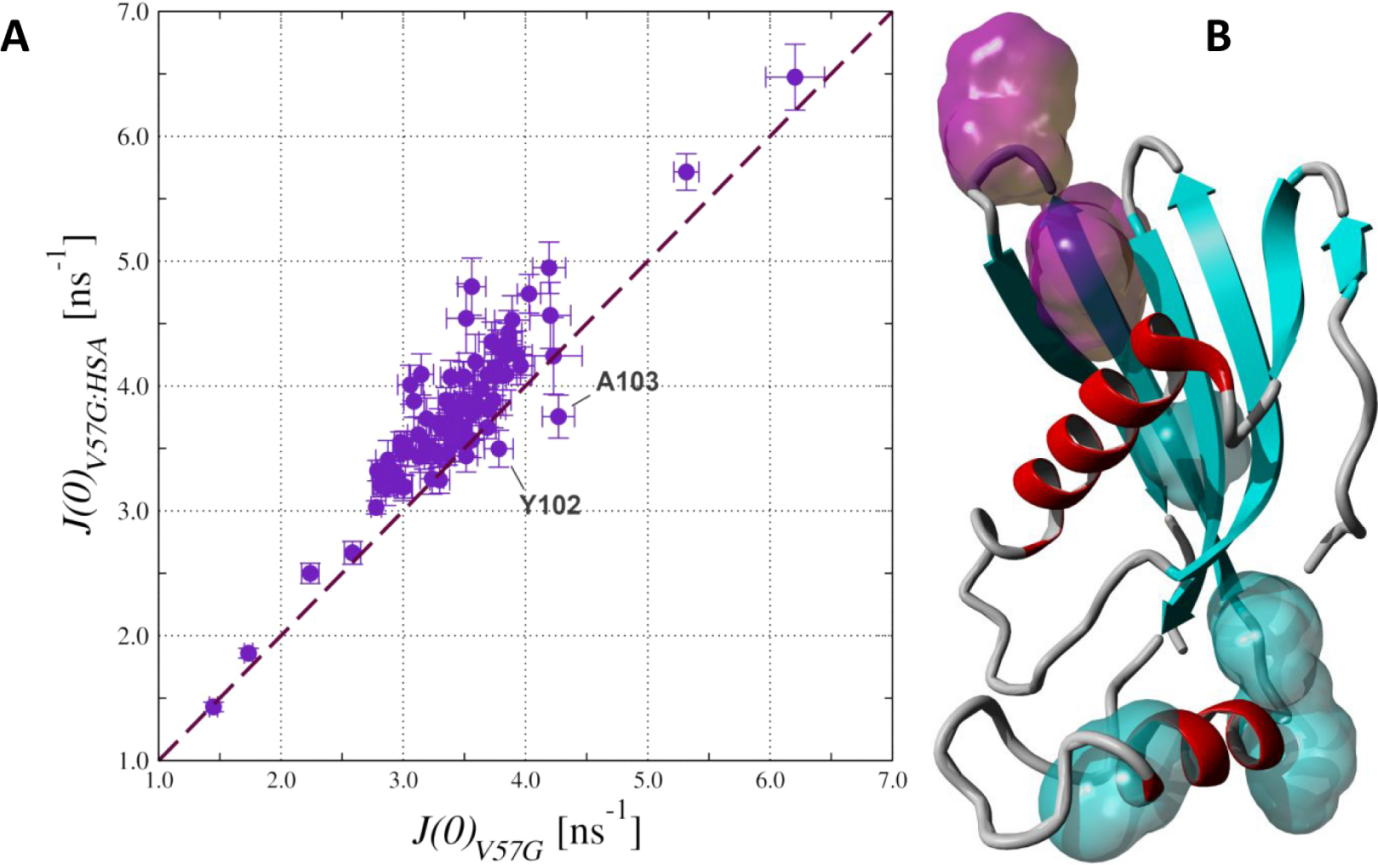
Comparison of the spectral density function (*J*(0)) values, evaluated for V57G HCC, in the absence and in the presence of HSA (A). 3D structure of the V57G HCC mutant in solution (B). Fragments displaying higher mobility in the low-frequency time frame, comprising residues Tyr102, Ala103, and Trp106, are highlighted in magenta, and residues with lower mobility are shown in cyan. Helices are shown in red.

## Discussion

Understanding the interactions between proteins present in physiological fluids is crucial to understanding the fluctuations in their concentrations related to the development and progression of various disease processes. This understanding facilitates the specification of their functions, interactions, and concentration ranges, thereby enhancing their utility as biomarkers for pathological processes occurring in the body and improving existing diagnostic procedures. Although both HSA and HCC proteins are present in blood plasma and cerebrospinal fluid in high concentrations, the possibility of their interactions was only speculated, and the complexation process between them has not yet been characterized. To study HSA–HCC direct interactions, we employed different complementary techniques such as affinity chromatography, mass spectrometry, MST, and ITC. It is worth mentioning that MST allows for the measurement of interactions between a labeled target (HSA) and an unlabeled binding partner (HCC), while ITC does not require the use of dyes.^33^ HSA and HCC exhibit varying concentrations in body fluids (e.g., for HCC, the highest concentration is noted in CSF^37^). Additionally, both proteins have the capacity to form oligomers in solutions.^38^ At neutral pH, which is characteristic of body fluids such as blood and cerebrospinal fluid, HCC predominantly exists in the monomeric form.^39^ On the other hand, HSA under these conditions is thought to be monomeric, although ca. 5% of it might be covalently dimerized.^40^ In further studies, we decided to use only monomeric forms of both proteins; HSA was purified by size exclusion chromatography to remove dimers and trimers, and in the case of HCC, we used the monomeric V57G variant to avoid dimer formation.

The initial results of mass spectrometry measurements confirmed complex formation; therefore, we decided to study it in more detail and determine the strength of these interactions using MST and ITC. Regardless of the method used, similar values of the dissociation constant for the HSA–HCC complex were obtained. The *K*_D_ value obtained from the MST method was 1.3 μM, while the dissociation constant determined by ITC was 1.15 μM. The obtained values of the dissociation constant confirm that the complex between both proteins is indeed formed, although its stability is not high. However, when these values are analyzed in the context of the relationship between binding affinity and interfacial buried surface area for a series of complexes of various proteins conducted by Chen et al.,^41^ it is worth noting that the potential interfacial buried surface area for the HSA–HCC system may be approximately 1000–1200 Å^2^. This suggests that such an interfacial area can be expected for the HSA–HCC complex.

Interestingly, Zhang et al.^42^ found that HCC can bind to another extracellular molecule – a glycosaminoglycan, heparan sulfate—at pH below 7, where HCC is positively charged (pI of HCC is 8.75). Furthermore, the *K*_D_ value for HCC binding to immobilized heparan sulfate, as probed by SPR, was in a range from 0.8 mM at pH 5.5 to 1.8 mM at pH 6.5. These numbers are in line with the *K*_D_ value obtained in this study for interaction with HSA, although the binding to the hexasaccharide fragment of heparan sulfate is mediated mainly by the positively charged N-terminal fragment and amino acids around His90 of HCC.

The binding site of the HCC–HSA complex was further investigated using NMR. The analysis of the obtained relaxation data indicated that HCC interacts with HSA via residues located in the unstructured C-terminus, mostly engaging the β-sheet motif encompassing amino acid residues 102–116 in the HCC sequence. Direct interactions were observed with the amino acid residues 101–103 of the HCC polypeptide chain, where the aromatic ring of tyrosine 102 is exposed for hydrophobic interaction with HSA. The association of HCC with the hydrophobic surface of HSA can destabilize HCC in the β-sheet structure formed by the 101–117 amino acid segment, as evidenced by an increase in dynamic behavior observed in NMR experiments.

HSA can form complexes with a variety of proteins, including glycoprotein 60, gp18, apolipoprotein B-100, IgG receptor FcRn large subunit p51, fibronectin type III, SPARC, etc.^15^ For some of these complexes, atomic structures can be found in the PDB database. Representative structures of HSA–protein complexes obtained by X-ray crystallography (PDB: 4HGM, 6ZL1, 2VDB, and 6M58) are presented in Figure 10. These proteins interact mostly through amino acids in the fragments spanning the HSA residues 220–235, 255–273, and 299–333 (see Figure 10) and a similar interaction area can probably be proposed for HCC. However, we do not have direct structural data confirming such an assumption.

**Figure 10:**
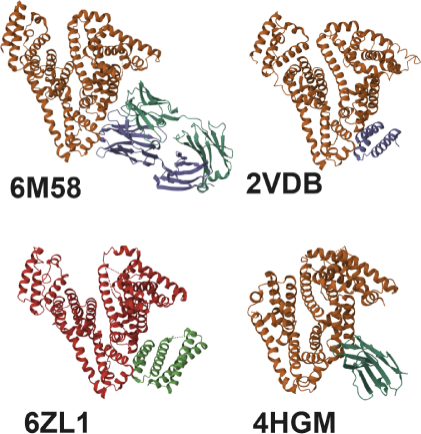
Comparison of the selected HSA complexes with proteins (antibody Fab SL335 ^43^ – PDB ID:6M58, S-naproxen and the GA module^44^ – PDB ID:2VDB, MCL-1 neutralizing alphabody CMPX-383B^45^ – PDB ID 6ZL1 and shark IgNAR variable domain^46^ – PDB ID: 4HGM). The images were generated using the Mol* viewer.^47^

It is tempting to speculate that HCC also binds to this region of HSA and that it serves as a nonspecific binding site for different proteinaceous cargos. However, the interactions of human serum albumin with other protein partners, characterized using protein crystallography, do not only concern the HSA sequence region between 210 and 340. HSA also forms a complex of different geometry with the neonatal Fc receptor (PDB ID: 4K71).^48^ In this case, the location of the interacting region in the HSA molecule is exactly on the opposite side of the molecule – in the area of domain III B.

The future studies should therefore focus on verification of this hypothesis. Additionally, since in pathological states HCC is known to form biologically inactive dimers, it is worth conducting similar studies but using the HCC dimer as the HSA ligand. If the HSA protein can bind not only to a monomer but also to a dimer of HCC, it may be a valid candidate for biosensor molecule detection of misfolded HCC. Such experiments are currently underway in our laboratory.

## Methods

### Materials

Human cystatin C wild-type (WT) protein was expressed in *Escherichia coli*, isolated, and purified as described previously.^39^ The monomeric V57G HCC variant was produced according to a previously described procedure.^27^ HCC samples, after purification, were lyophilized and stored at −80 °C. Human serum albumin was purchased from Sigma-Aldrich, (USA), #A8763. The HSA sample before experiments, was purified by size exclusion chromatography (Superdex 200 Increase 10/300 GL, Cytiva, Sigma-Aldrich, USA) to avoid dimers. The samples were routinely checked for monodispersity using dynamic light scattering (DLS) and a Litesizer 500 system (Anton Paar GmbH, Graz, Austria).

### Microscale Thermophoresis

HCC samples intended for microscale thermophoresis (MST) experiments were first dissolved in ice-cold PBS, pH 7.4 (PBS buffer, BioShop, Canada, #PBS404.100), and then centrifuged for 20 min (20 000 *g*, 4 °C). HSA samples were dissolved and centrifuged analogously and next subjected to an additional gel filtration step as described above. Monomeric HSA was labeled with the Monolith NT Protein Labeling Kit RED-NHS (Amine Reactive) according to the manufacturer’s protocol. Simultaneously, a titration series of 16 dilutions of HCC (unlabeled binding partner) was prepared. A molecular interaction experiment and measurements of the dissociation constant (*K*_D_) were performed at room temperature in standard capillaries (K002), according to the standard protocol (Monolith NT.115, NanoTemper, Germany). The experiment was performed in triplicate.

### Isothermal Titration Calorimetry

Isothermal titration calorimetry (ITC) experiments were carried out using a Malvern MicroCal PEAQ-ITC system (Malvern Panalytical, Malvern, UK) at 25.2 °C. The samples for ITC were prepared analogously to those for MST measurements. Before the experiment, HCC and HSA samples were dialyzed in one beaker against degassed PBS at 4 °C overnight. The instrument syringe was filled with HSA solution at 300 μM, and the cell was filled with HCC solution at 30 μM. The experiment consisted of a first 0.4 μL injection followed by 14.3 μL injections of HSA solution into the cell containing HCC solution. Integration of the power peaks (μcal/s) provided the heat released upon binding of HSA to HCC. The calculations were performed by using MicroCal PEAQ-ITC Analysis Software. Baseline-adjusted data were fitted using a single set of identical site binding model, which yields the number of binding sites (*n*), dissociation constant (*K*_d_), and Δ*H*. Using the obtained *K*_d_ value, the thermodynamic parameters characterizing HSA–HCC interactions (Δ*G* and *T*Δ*S*) were calculated.

### Single Set of Identical Site Model Description

Binding constant *K*_d_ for the model of ligand (HSA in the syringe) binding to *n*-independent identical sites in the macromolecule (HCC in the cell) is determined as follows: $K_{d}=\frac{\theta }{\left(1-\theta \right)\left[HSA\right]}$ , where θ – fraction of sites occupied by the HSA, and HSA_t_ = [HSA] + *n*θ­[HCC]_t_

The total *Q* content in a solution of volume *V*_o_ at a fraction θ of the occupied total binding sites is

$$Q=n\theta HCC_{t}\Delta HV_{o}$$

Linking all above expressions gives the final equation for *Q*:

$$Q=\frac{nHCC_{t}\Delta HV_{0}}{2}\left[1+\frac{HSA_{t}}{nHCC_{t}}+\frac{1}{nK_{d}HCC_{t}}-\sqrt{{\left(1+\frac{HSA_{t}}{nHCC_{t}}+\frac{1}{nK_{d}HCC_{t}}\right)}^{2}-\frac{4HSA_{t}}{nHCC_{t}}}\right]$$

Where:

*n* – number of sites

HCC_t_ – concentration of HCC

HSA – free HSA concentration

HSA_t_ – total HSA concentration

*V*_o_ – initial volume of the cell

With the correction for the effect of displaced volume, given by the equation:

$$\Delta Q\left(i\right)=Q\left(i\right)+\frac{dV_{i}}{V_{o}}\left[\frac{Q\left(i\right)-Q\left(i-1\right)}{2}\right]-Q\left(i-1\right)$$

Where:

Δ*Q*(*i*) – corrected heat released upon *i*-th injection

*Q*(*i*) – *Q*(*i*) calculated using the equation for *Q*

d*V*_i_ – increase in cell volume after *i*-th injection

*V*_o_ – initial volume in the cell

The description presented above is based on the procedure adopted from ITC data analysis in Origin Tutorial Guide, ver 7.0 - January 2004 (MicroCal, LLC, Northampton, MA, USA).

### Preparation of the Microcolumn with Immobile HSA

To prepare columns for affinity chromatography experiments, 50 mg of CNBr-activated Sepharose (Sigma-Aldrich) was incubated to swell in 1 mM HCl for 30 min, according to the manufacturer’s recommendations. Next, the Sepharose was washed with 2 mL of H_2_O and 5 mL of binding buffer (0.5 M NaCl, 0.1 M NaHCO_3_, pH 8.3). 100 μg portion of HSA was suspended in 100 μL of binding buffer, applied to the media, and incubated with agitation at room temperature for 2 h. In the next step, unbound HSA molecules were removed with 5 mL of binding buffer. Unbound groups on the Sepharose were blocked by incubation in a blocking buffer (1 M ethanolamine) for 2 h with stirring at room temperature. The blocking buffer was removed by washing four times with alternating 5 mL of binding buffer and 5 mL of wash buffer (0.1 M CH_3_COONa, 0.5 M NaCl). In parallel, a control column was prepared in which the HSA binding step was replaced by incubation in blocking buffer. All columns were stored in 1 M NaCl at 4 °C.

### Affinity Chromatography

Human cystatin C was applied onto an HSA-Sepharose column equilibrated in PBS buffer (pH 7.4) and incubated for 2 h at room temperature. The unbound material was removed by washing with 80 mL of PBS. The first milliliters of the washing solution (supernatant) and the last milliliter of the washing solution (last wash fraction) were collected. HCC molecules that formed the complex with the immobilized HSA were dissociated using 2 × 500 μL of 0.1% aqueous trifluoroacetic acid (TFA, elution fraction). All three fractions—supernatant, last wash, and elution—were collected, desalted using C4 ZipTip (Merc Millipore), and analyzed using MALDI TOF/TOF 5800 (Sciex, Germany). Mixtures containing 0.6 μL of sample and 0.6 μL of 10 mg/mL 2,5-dihydroxybenzoic acid matrix (DHB) were applied to the MALDI-MS analysis plate. After crystallization, the plate was placed in a MALDI TOF/TOF mass spectrometer, and mass spectra were collected in linear mode (Linear Middle Mass Positive) in the mass range of 5000–30 000 *m*/*z*. The registered spectra were analyzed with DataExplorer software.

### In-Solution Analysis of the HSA Complex with HCC Using ESI MS High-Resolution Mass Spectrometry

A 1:1 mass ratio of HSA and HCC (concentration: 1 μg/μL) in 50 mM ammonium formate was prepared. Analysis of the solution, dispensed with a syringe pump, was performed using a TripleTOF 5600+ (Sciex) mass spectrometer equipped with a DuoSpray Ion Source (ion source parameters: ISVF = 5500 V; TEM = 0 °C; CUR = 30 psi; GS1 = 30 psi; GS2 = 0 psi; DP = 180 V; EC = 10). The test was carried out in the positive ion mode for 5 min, during which particles with masses in the range of 400–3500 Da were collected (accumulation time: 0.99 s). The spectrometer was operated with the Intact Protein Mode function activated. Analysis of the recorded spectra and mass reconstruction were performed in PeakView 2.2.0.11391 software (Sciex) using the Bio Tool Kit.

### Sample Preparation for NMR Spectroscopy

The HCC V57G nuclear magnetic resonance (NMR) sample for the relaxation measurements was prepared by dissolving V57G HCC in PBS buffer prepared with a 90%/10% H_2_O/D_2_O mixture. The HSA was prepared under the same conditions. The pH value was stabilized at 7.0 (uncorrected value). The V57G HCC complex with HSA was obtained by mixing the solution of V57G HCC with HSA to obtain equimolar concentrations (around 0.3 mM) of both proteins.

### ^15^N Relaxation Measurements

The NMR experiments were performed at 298 K on Varian Inova 500 and Agilent DDR2 NMR spectrometers operated in magnetic fields of 11.7 and 18.8 T, respectively. Both spectrometers were equipped with at least three channels, ^1^H/^13^C/^15^N triple resonance probe-heads with inverse detection, a *z* gradient Performa-IV module, and a temperature control unit. The acquired data were referenced indirectly to an external sodium 2,2-dimethyl-2-silapentane-5-sulfonate (DSS) standard using the coefficient value of Ξ = 0.101329118 for the ^15^N dimension.^49^ Collected NMR data were processed with the NMRPipe software^50^ and analyzed with the NMRFAM-Sparky software.^51^

The experimental ^15^N relaxation data sets, including longitudinal (*R*_1_) and transverse (*R*_2_) relaxation rates, together with ^1^H–^15^N NOE, were collected at a magnetic field of 11.7 T (^1^H resonance frequency – 500.606 MHz) utilizing a Varian Inova 500 NMR spectrometer. Pulse sequences were taken from BioPack software (Agilent, USA), which were adapted from previously published experiments.^51^ ^15^N *R*_1_ data sets were acquired with nine evolution times: 10, 90, 170, 290, 410, 550, 690, 850, and 1010 ms. ^15^N *R*_2_ relaxation rates were measured according to the Carr–Purcell–Meiboom–Gill (CPMG) pulse train with a 650 μs refocusing time. *R*_2_ values were evaluated based on eight delays – 10, 30, 50, 70, 90, 110, 130, and 150 ms. The recycling delays for *R*_1_ and *R*_2_ experiments were defined as 3.5 s. The ^1^H–^15^N NOE values were obtained as the ratio between peak amplitudes detected in saturated (*I*_sat_), and reference (*I*_ref_) experiments.^52^ The recycle delays and saturation times of ^1^H magnetization in both NOE experiments were kept at 6 s.

### ^15^N Relaxation Data Analysis

The ^15^N *R*_1_ and *R*_2_ relaxation rates were calculated for the 82 amide nitrogen atoms with a two-parameter model (single exponential decay) evaluated by fitting peak amplitudes with monoexponential decay in the RELAX software (version 4.0.3).^53^ Errors for the relaxation parameters were calculated with 200 Monte Carlo simulations based on the signal-to-noise ratio established with NMRFAM-Sparky software. The evaluated experimental data sets were analyzed with the spectral density mapping (SDM) approach, which provided the spectral density of molecular dynamics processes in the protein’s backbone amide groups at the frequencies of 0, ω_N_, and 0.87 ω_H_.^54^
